# Plasma cfDNA predictors of established bacteraemic infection

**DOI:** 10.1099/acmi.0.000373

**Published:** 2022-06-14

**Authors:** Nadezda Urosevic, Adam J. Merritt, Timothy J. J. Inglis

**Affiliations:** ^1^​ School of Biomedical Sciences, Faculty of Health & Medical Sciences, The University of Western Australia, Nedlands, WA, Australia; ^2^​ School of Medicine, Faculty of Health & Medical Sciences, The University of Western Australia, Nedlands, WA, Australia; ^3^​ Department of Microbiology, PathWest Laboratory Medicine, QEII Medical Centre, Nedlands, WA, Australia

**Keywords:** plasma cell-free DNA, bacteraemia, sepsis, nucleosomal cfDNA, microbial cfDNA, C-reactive protein

## Abstract

**Introduction**. Increased plasma cell-free DNA (cfDNA) has been reported for various diseases in which cell death and tissue/organ damage contribute to pathogenesis, including sepsis.

**Gap Statement**. While several studies report a rise in plasma cfDNA in bacteraemia and sepsis, the main source of cfDNA has not been identified.

**Aim**. In this study, we wanted to determine which of nuclear, mitochondrial or bacterial cfDNA is the major contributor to raised plasma cfDNA in hospital subjects with bloodstream infections and could therefore serve as a predictor of bacteraemic disease severity.

**Methodology**. The total plasma concentration of double-stranded cfDNA was determined using a fluorometric assay. The presence of bacterial DNA was identified by PCR and DNA sequencing. The copy numbers of human genes, nuclear *β globin* and mitochondrial *MTATP8*, were determined by droplet digital PCR. The presence, size and concentration of apoptotic DNA from human cells were established using lab-on-a-chip technology.

**Results**. We observed a significant difference in total plasma cfDNA from a median of 75 ng ml^−1^ in hospitalised subjects without bacteraemia to a median of 370 ng ml^−1^ (*P*=0.0003) in bacteraemic subjects. The copy numbers of nuclear DNA in bacteraemic also differed between a median of 1.6 copies µl^−1^ and 7.3 copies µl^−1^ (*P*=0.0004), respectively. In contrast, increased mitochondrial cfDNA was not specific for bacteraemic subjects, as shown by median values of 58 copies µl^−1^ in bacteraemic subjects, 55 copies µl^−1^ in other hospitalised subjects and 5.4 copies µl^−1^ in healthy controls. Apoptotic nucleosomal cfDNA was detected only in a subpopulation of bacteraemic subjects with documented comorbidities, consistent with elevated plasma C-reactive protein (CRP) levels in these subjects. No bacterial cfDNA was reliably detected by PCR in plasma of bacteraemic subjects over the course of infection with several bacterial pathogens.

**Conclusions**. Our data revealed distinctive plasma cfDNA signatures in different groups of hospital subjects. The total cfDNA was significantly increased in hospital subjects with laboratory-confirmed bloodstream infections comprising nuclear and apoptotic, but not mitochondrial or bacterial cfDNAs. The apoptotic cfDNA, potentially derived from blood cells, predicted established bacteraemia. These findings deserve further investigation in different hospital settings, where cfDNA measurement could provide simple and quantifiable parameters for monitoring a disease progression.

## Introduction

The potentially life-threatening disease known as sepsis is the clinical manifestation of a predominantly bacterial bloodstream infection. Escalating antibiotic resistance in the most commonly encountered bacterial causes reduces the efficacy of sepsis treatment and patient survival [1–3]. The best means of preventing sepsis progression is by stopping its progression by early detection and treatment of the bloodstream infection [4, 5]. Despite improved methods that confirm bacteraemia, culture-based microbiology methods are cumbersome and do not deliver actionable results early enough in bacteraemic infection to intervene with effective antimicrobial therapy. The variety and complexity of physiological reactions to blood infections have defied attempts to identify a reliable predictive biomarker for early sepsis. Previously, the concept of a systemic inflammatory response syndrome (SIRS), arising from overstimulation of the immune system by the infectious agent, was used as a surrogate for sepsis and applied to clinical management [6, 7]. Despite describing the pathophysiology of sepsis, SIRS was omitted from the consensus definitions of sepsis by the Society of Critical Care Medicine and the European Society of Intensive Care Medicine task force, due to SIRS’s lack of specificity, poor utility in disease monitoring, and treatment failures with anti-inflammatory agents [8, 9]. The Third International Consensus Definition (Sepsis-3) omitted SIRS altogether, dismissing it as a non-critical determinant of survival in sepsis [10, 11]. Features that discriminate sepsis from bacteraemia are ‘dysregulated host responses to infection’ resulting in ‘life-threatening organ dysfunction’, reflected in elevated qSOFA scores [12]. This distinction between bacteraemia and sepsis is not widely accepted, and indicates a need for additional sepsis markers beyond the demonstrable presence of bacteria in the bloodstream [13].

Non-specific blood biomarkers that may predict sepsis include procalcitonin, C-reactive protein and sepsin [14, 15]. Since the Sepsis-3 definition emphasises on dysregulated responses to infection that cause tissue damage and fatal organ dysfunction [10, 11], it is plausible that these responses accompany changes in other blood components. Blood cells show remarkable fluctuations in number, morphology and distribution during infection including sepsis, so that changes in vital organ activity may be mirrored by alterations in blood constituents. One of the blood components that responds to changes in blood cell viability and peripheral tissue damage is extracellular or cell-free DNA (cfDNA), which is released into the circulation in response to physiological and pathological stimuli [16]. The composition, amount and timing of cfDNA have been studied in a variety of conditions as a potential sepsis biomarker, if used in combination with other disease-defining parameters [17]. However, no comparative analyses of cfDNA derived from different sources have been performed in bacteraemia illness.

Here we report our findings on total plasma cfDNA content and copy numbers of human nuclear and mitochondrial genes released in the plasma of hospitalised subjects with confirmed bloodstream infections. These records were supplemented with data on nucleosome-size nuclear cfDNA in all subjects. We also studied the contribution of bacterial DNA to plasma cfDNA in bacteraemic subjects. Taken together, these analyses provide a comprehensive tool to monitor the severity of host responses to bloodstream infections.

## Methods

### Study subjects

This study was performed on plasma isolated from EDTA blood specimens routinely collected for standard clinical laboratory procedures at a single tertiary hospital campus (Sir Charles Gairdner Hospital, Nedlands, Western Australia). This observational series included specimens from hospital-admitted subjects who had only one blood culture (BC) set collected for investigation of suspected blood infection, subjects with multiple BC sets, and with no BC collection during their hospital admission.

The number of EDTA blood collections varied during each bacteraemic episode (defined as 0 to ±30 h from the time of positive BC) between single and multiple. We therefore used a single EDTA sample from each subject according to proximity to the BC collection and proximity to the peak of response to infection corresponding to the highest increase in plasma analytes. These two times were the same in the majority of subjects so that only small differences in median values were observed between the two (shown in Results). Healthy adult volunteers with a range of age and gender were used as a source of control specimens.

### Plasma cfDNA analyses

To obtain plasma, EDTA blood was centrifuged at 800 ***g*** for 10 min at room temperature followed by 5 min centrifugation at 15000 ***g*** to remove cellular debris [18]. Plasma supernatant was either frozen at −80 °C or used immediately to extract fresh cfDNA with a QIAamp DNA Blood Mini Kit (Qiagen, MD) as described previously [18]. The concentration of total plasma cfDNA was determined using a Qubit dsDNA HS Assay Kit and a Qubit 2.0 Fluorometer (Life Technologies, CA). A qualitative analysis of total plasma cfDNA and determination of the concentration of nucleosome-size cfDNA were performed using an Agilent DNA 12000 Kit and Agilent 2100 Bioanalyzer (Agilent Technology, CA).

### Droplet digital PCR of mitochondrial and nuclear genes

Copy numbers of nuclear and mitochondrial cfDNA in plasma were determined by duplex PCR using a QX100 Droplet Digital PCR (ddPCR) System (Bio-Rad, CA). These duplex PCR assays were performed with ddPCR Supermix for Probes (Bio-Rad, CA) using the following primers and probes for the nuclear *β globin* (*GLOB*) and mitochondrial *ATPase 8* (*MTATP 8*) genes as follows: GLOB-524F: 5′-GGC ATG TGR AGA CAG AGA-3′; GLOB-579R: 5′-SAS AGA GAG TCA GTG CCT AT-3′ and TaqMan GLOB probe: 5′-LightCycler-640- AGA AAC CCA AGA GTC-BHQ3-3′ (Roche, Australia) [18]; MTATP8-F: 5′-AAT ATT AAA CAC AAA CTA CCA CCT ACC-3′; MTATP8-R: 5′-TGG TTC TCA GGG TTT GTT ATA-3′ and MTATP8 probe: 5′-VIC-CCT CAC CAA AGC CCA TA-MGBNFQ-3′ (Roche, Australia) [19]. The final concentration for primers was 900 nM, and for probes 250 nM, respectively. The cycling conditions (using a Thermal Cycler C1000 Touch, Bio-Rad) were as follows: enzyme activation for 10 min at 95 °C, 40 cycles of denaturation at 94 °C for 30 s and annealing/extension at 60 °C for 1 min with 2 °C/sec ramp setting, and a final hold at 98 °C for 10 min. One microlitre plasma cfDNA extract per reaction per sample was used in a duplex ddPCR reaction. After this was completed, positive and negative droplets in each sample and copy numbers of *β globin* and *MTATP 8* genes were determined using a QX100 Droplet Reader and QuantaSoft Software (Bio-Rad, CA), respectively.

### Bacterial PCR

Bacterial PCR was performed on plasma cfDNA extracted using a ThermoFisher Scientific KingFisher Flex instrument as previously described [20]. Plasma DNA was extracted from 200 µl aliquot of EDTA plasma and eluted in 50 µl of elution buffer. Five PCR reactions were set up with 8 µl of plasma DNA extract and 12 µl of master mix for each of five sets of universal bacterial 16S primer pairs as follows: Set 1 – RWO-1F 5′-AACTGGAGGAAGGTGGGGAT-3′, DG-74R 5′- AGGAGGTGATCCAACCGCA-3′ [21]; Set 2 – BACT-16F 5′-GAAGAGTTTGATCATGGCTCAG-3′ [22], BACT-575R 5′- GTATTACCGCGGCTGCTGGCAC-3′ [23]; Set 3 – BACT-27F 5′-AGAGTTTGATCMTGGCTCAG-3′ [22], BACT-805R 5′-GACTACCAGGGTATCTAATCC-3′ [24]; Set 4 – BACT-13B 5′-CGGGATCCCAGGCCCGGGAACGTATTCAC-3′ [25], BACT-91E 5′-GGAATTCAAACGAATTGACGGGGGC-3′ [26]; Set 5–554-REV 5′-GTGCCAGCAGCCGCGGTAATAC-3′, RWO1-REV 5′-ATCCCCACCTTCCTCCGGTT-3′. The PCR master mix consisted of 2.5 mM MgCl_2_ (Life Technologies, Mulgrave, Australia), 0.2 mM each nucleoside triphosphate (Fisher Biotec, Wembley, Australia), one unit of HyperTaq polymerase (Nature Technology, Lincoln, USA), 1 x HyperTaq buffer (Nature Technology, Lincoln, USA), 0.01 % v/v bovine serum albumin (Sigma Aldrich) and 0.2 µM of each primer. Amplification was performed using the following cycling parameters: 95 °C for 5 min, 45 cycles of 94 °C for 30 s, 55 °C for 30 s and 72 °C for 45 s followed by a final extension of 72 °C for 7 min. PCR products were separated by gel electrophoresis in 2.5 % agarose gels with GelRed Dye (Biotium, Fremont, USA) and visualised on a UV transilluminator. PCR products with an amplicon were purified using illustra ExoStar 1-Step (GE Healthcare, Chicago, IL, USA) according to manufacturer’s instructions and sequenced using ABI BigDye version 3.1 sequencing chemistry (Applied Biosystems, Foster City, CA, USA) on an ABI 3730 sequencer (Applied Biosystems) in the forward and reverse direction following bead-based clean-up with Axygen AxyPrep MAG DyeClean beads (Corning, Amsterdam, The Netherlands). Resulting sequences were manually checked for base call errors (Chromas version 2.4.3; Technelysium, South Brisbane, Qld, Australia) and compared to sequences in the GenBank database using blast [27].

### Plasma CRP and subjects’ demographic data

Plasma C-reactive protein (CRP) levels were determined in the clinical diagnostic laboratory for subjects with suspected bloodstream infection and inflammation. We obtained the CRP data from the clinical laboratory record and used it to measure infection severity in the absence of the qSOFA scores.

Plasma cfDNA extractions were performed as reported previously [18] without prior knowledge of subjects’ demographic or clinical data, BC status and interval between positive BC and EDTA blood collections. Subjects’ demographic data including age and sex, and limited clinical data such as CRP levels, white blood cell (WBC) counts and comorbidities relevant to sepsis, were obtained from the clinical laboratory record. Underlying medical conditions were divided into several categories as follows: death >sepsis/SIRS>stroke/myocardial infarction >renal failure >cancer/tumours>febrile/surgery>chest/respiratory. We also noted any admission to the emergency department (ED) or intensive care unit (ICU), and documented evidence of non-vital organ infections to further describe their condition. Suspected blood culture contamination documented in the laboratory information system was recorded.

### Statistical analyses

Statistical analysis was performed using statistical software (Prism 9, GraphPad, San Diego, CA) and comprised descriptive statistics and the Mann-Whitney U test.

## Results

### Study participant characteristics

**Fig. 1.**
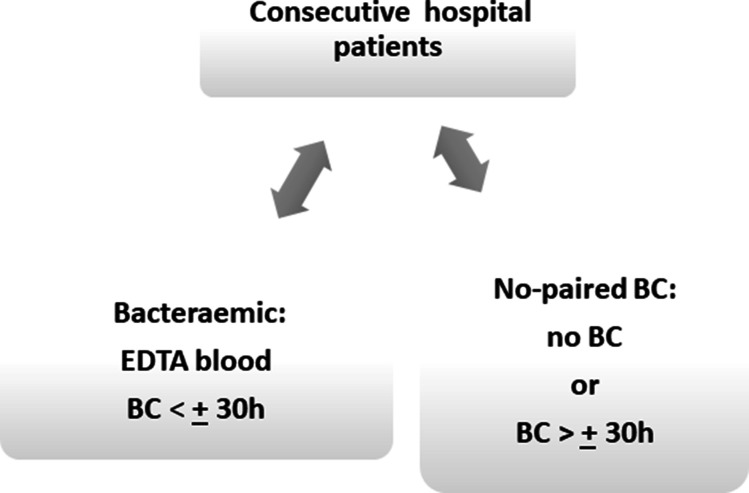
A diagram of hospital subject classification. Consecutive hospital admissions with suspected bloodstream infection were grouped as bacteraemic and no paired BC according to the relative timing of BC evidence for bloodstream infection to EDTA blood collection. The bacteraemic group comprised subjects with EDTA blood samples collected within 30 h of a positive BC. No paired BC subjects had negative BC, no BC collection during their hospital stay or had their EDTA blood collected >30 h before or after BC collection.

Samples were obtained from 63 subjects, a consecutive series of subjects admitted to Sir Charles Gairdner Hospital, Nedlands, Western Australia as reported in the previous study [18]. All subjects had suspected bloodstream infection, as evidenced by a blood culture collection. Since the interval between positive BC and EDTA blood collections varied, ranging from 0 to more than 100 h (median interval 24 h and average 32 h), we used a 30 h limit to divide subjects into two groups, summarized in Fig. 1. Out of 63 subjects in the study, 49 had their EDTA blood collected within a 30 h interval before and after positive BC. These were designated bacteraemic (Table 1, BAC subjects). A second group comprised 14 subjects who either did not have any BC collection within ±30 h of EDTA blood collection, or who had no BC collection during their entire hospital stay (Table 1, NBC subjects). Accordingly, there were 49 bacteraemic subjects and 14 no paired BC subjects without corresponding EDTA blood specimen, and nine healthy control subjects in the study (Table 1). The demographic and clinical details are presented in Table 2. For all subjects, only a single EDTA blood specimen was used for further analyses, as described below. The bacterial species detected in positive BCs and their clinical significance are listed in Table 3.

**Table 1.** Subject inclusion according to blood culture collections

Subject category	No	Inclusion criteria
BAC*	49	BC+
NBC†	14	BC-/no BC
Healthy	9	no disease

*BAC – Bacteraemic subjects - blood culture (BC) and EDTA Blood (EB) collected within 30 h.

†NBC – No paired BC patients with negative BC or no BC collected within ±30 h of EB collection.

**Table 2.** Subject demographic and clinical data

		Bacteraemic	No paired BC	Healthy
**Cases**	Number	49	14	9
	Min, Max	22, 93	41, 84	22, 58
**Age** (yr)	Ave	64.06	63.07	45
	Median	65	66	50
**Gender**	F	23 (47 %)	5 (36 %)	3
	M	26 (53 %)	9 (64 %)	6
	Min, Max	0.1, 48.8	3.7, 17.4	
**White Blood Cells**	Ave	11.5	8.3	
(×10^3^ µl^−1^)	Median	9.6	7.9	
	Min, Max	0.9, 490	3.7, 66	
**CRP** (mg l^−1^)	Ave	151	28.7	
	Median	120	32	
**BC Collection**	Single	14 (29 %)		
	Multiple (2-8)	35 (71 %)		
	Gram +	33 (67 %)		
**Bacteria in BC**	CoNS*	18 (37 %)		
	Gram -	14 (29 %)		
	Mixed	2 (4 %)		

*CoNS – coagulase negative staphylococci.

**Table 3.** The list of bacterial isolates from BC

Clinically significant bacteria			
Likely	No	Possible*	No
*S. aureus*	2	*S. epidermidis*	8
*S. pneumoniae*	4	*S. capitis*	4
*S. pyogenes*	1	*S. salivarius*	2
* Enterococcus* spp.	2	*S. infantarius*	1
* Enterobacter* spp.	1	*S. mitis*	1
* Acinetobacter* spp.	1	*CoNS*	9
*S. dysgalactiae*	1		
*E. coli*	6		
*K. pneumoniae*	3		
*P. aeruginosa*	2		
*P. mirabilis*	1		

*Commensal bacteria or skin flora.

### Plasma cfDNA composition

**Fig. 2.**
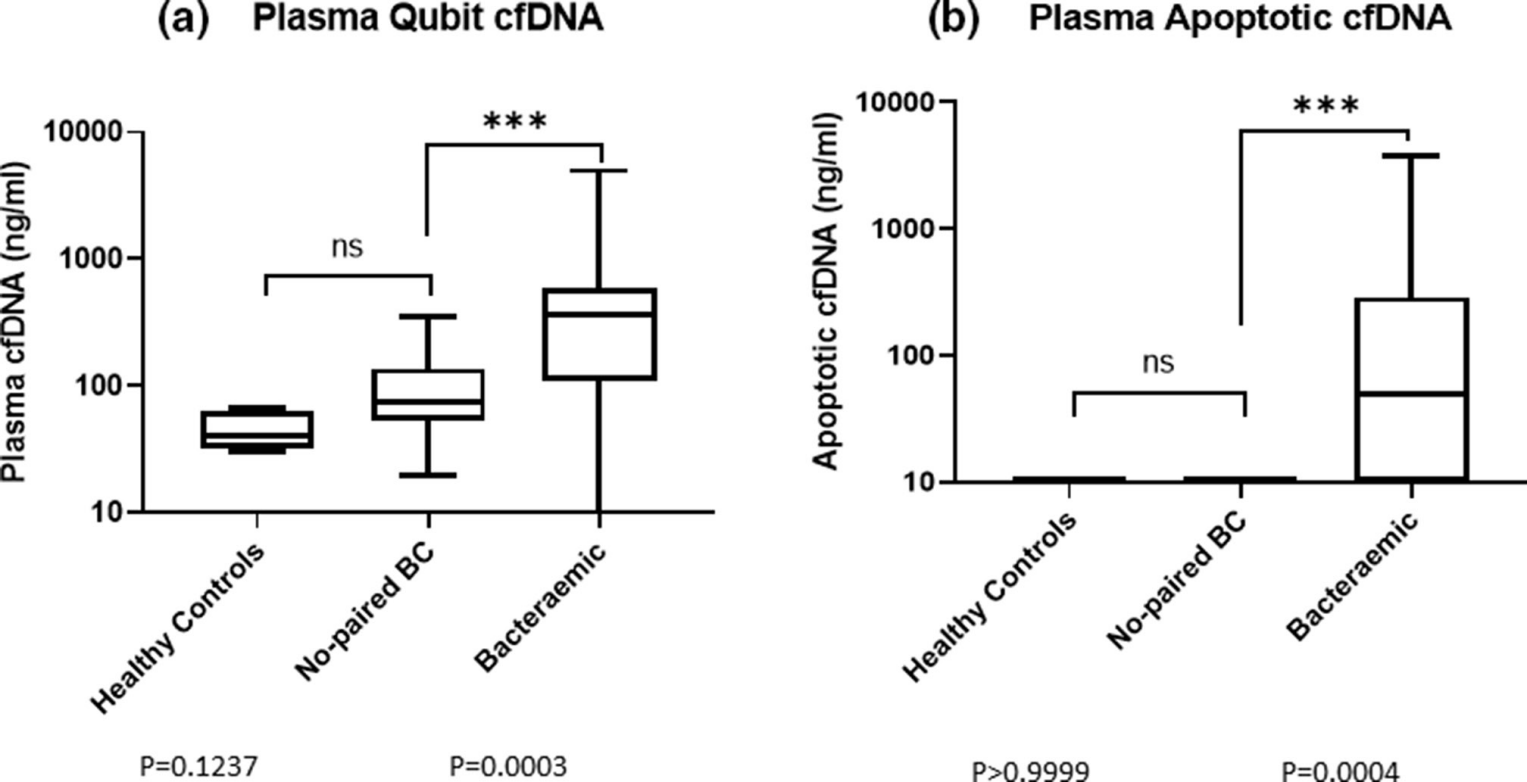
Analysis of total plasma dsDNA and apoptotic DNA between different subject groups. **a**) The total amount of dsDNA extracted from bacteraemic subjects’ plasma, no paired BC subjects and healthy controls was determined by Qubit. **b**) Following a microfluidic DNA gel electrophoresis of total plasma dsDNA, a single discrete DNA band in the range of 160 to 192 base pairs (bp) was identified and measured in different groups of subjects and healthy controls by Bioanalyzer. The statistical analyses in A and B were performed with GraphPad Prism 9. Variance was determined by the Mann Whitney U test. EDTA blood samples most proximal to BC with the highest plasma CRP levels were used in the analysis.

At the beginning of the study, we used direct fluorometric measurements to determine total double-stranded (ds) DNA by Qubit and nucleosomal cfDNA derived from apoptotic blood cells by Bioanalyzer, subsequently referred to as apoptotic cfDNA. According to the Bioanalyzer measurements, the minimum size of apoptotic cfDNA detected in plasma from bacteraemic subjects was 160 base pairs (bp) and maximum 192 bp with average and median values at 168 bp. There was no significant difference in median amounts of total plasma cell-free dsDNA (40 ng ml^−1^ for healthy controls vs 75 ng ml^−1^ for no paired BC) and apoptotic cfDNA (0 in both) between healthy controls and no paired BC subjects, while in the plasma of bacteraemic subjects there were significant increases (median total cfDNA 370 ng ml^−1^, *P*=0.0003; Fig. 2a and 250 ng ml^−1^, *P*=0.0007, Fig. S1 (available in the online version of this article); median apoptotic cfDNA 50 ng ml^−1^, *P*=0.0004; Fig. 2b and 20 ng ml^−1^, *P*=0.001, Fig. S1b by Mann-Whitney two-tailed U test) dependent on the sampling criteria applied as described above, in both total and apoptotic cfDNA during the bacteraemic episode (Fig. 2).

**Fig. 3.**
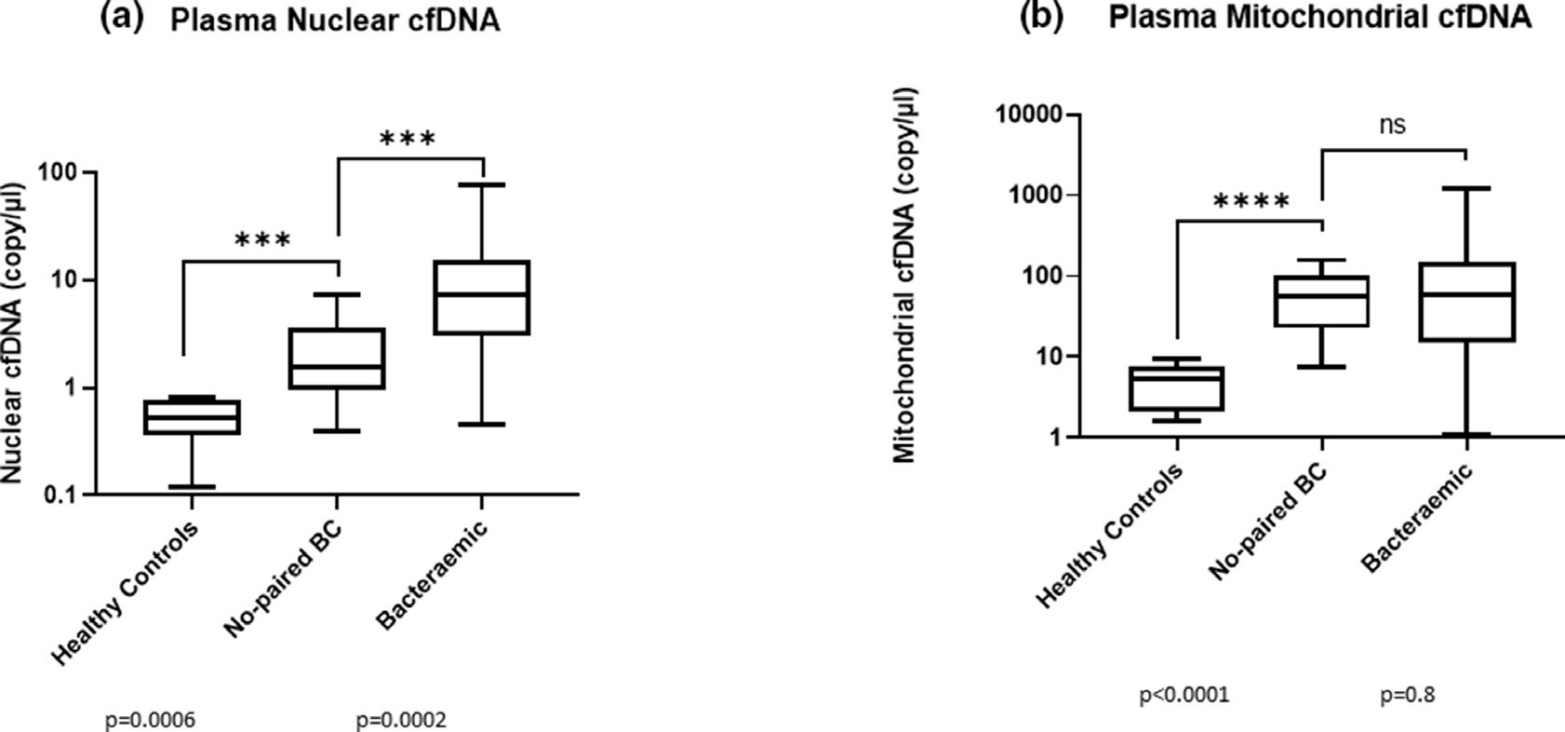
Copy number of nuclear *β globin* and mitochondrial *MTATP8* genes by ddPCR. **a**) The copy number of the nuclear *β globin* gene in plasma cfDNA of healthy controls and two groups of hospitalised subjects, no paired BC and bacteraemic, shown here using box and whisker plots (GraphPad Prism 9). **b**) The copy number of the mitochondrial *MTATP8* gene was simultaneously determined for the same healthy controls and no paired BC/bacteraemic subjects and plotted using box and whisker plots (GraphPad Prism 9). Variance was determined by the Mann Whitney U test. EDTA blood samples of hospital subjects most proximal to BC with the highest plasma CRP levels and EDTA blood samples from only four healthy controls were used in the analysis.

Plasma cfDNA composition and content varied greatly in hospital-admitted subjects creating difficulty measuring extremely low and very high DNA concentrations by a single method [18]. To overcome this, a droplet digital PCR was applied to determine the absolute copy numbers of the nuclear *β globin* and mitochondrial *MTATP8* genes in plasma cfDNA in a single duplex ddPCR reaction per specimen. As shown in Fig. 3, healthy people had very low concentrations of both nuclear and mitochondrial cfDNAs in blood plasma, while these components are significantly increased in no paired BC subjects on hospital admission (Mann-Whitney U test, *P*=0.0006 and *P*<0.0001, respectively). Nuclear cfDNA represented by the copy number of *β globin* gene showed a further significant increase in the bacteraemic group of subjects in comparison to no paired BC subjects (Fig. 3a, Mann-Whitney, *P*=0.0002;Fig. S2a, *P*=0.0004). Mitochondrial cfDNA showed similar increases in both groups of hospitalized subjects, and no additional increases in subjects with confirmed bacteraemia (Fig. 3b and S2b).

Remaining EDTA plasmas from 15 bacteraemic and one no paired BC subjects collected at various intervals relative to positive BC were subjected to routine DNA extractions and bacterial PCR analyses using five sets of universal bacterial 16S primer pairs (Table 4). No bacterial DNA was detected in any plasma sample analysed including those with high cfDNA content sampled within a critical ±30 h period relative to positive BC. There was a weak band detected only in one sample with one set of 16S primers at 27 h post-positive BC (Table 4). Nucleotide sequencing revealed a sequence corresponding to a common skin commensal bacterium rather than to a pathogenic bacterium detected by the routine BC analysis (data not shown).

**Table 4.** Bacterial 16S PCR results on plasma samples from bacteraemic and no paired BC subjects at various time points relative to positive blood culture. n.d., Not detected; w.b., weak band.

Bacterium/	>-30h		−30h<0<30h		>30 h	
Subject	Qubit (ng µl^−1^)	16S PCR	Qubit (ng µl^−1^)	16S PCR	Qubit (ng µl^−1^)	16S PCR
Sepi/Scap-2	1.79	n.d.	0.54	n.d.		
CNS/Efu-3			0.06	n.d.		
Kpn-3^#^			4.98	n.d.		
Entc-6*			0.27/0.66†	n.d./n.d.‡		
Psa-12			0.16	n.d.	0.25	n.d.
Spn-13	0.5	n.d.	2.28	n.d.		
MRSA/MSSA-16			1.16/0.24†	n.d./n.d.‡		
No-paired BC-17*					0.08	n.d.
Sepi-23			0.39/0.49†	n.d./n.d.‡		
Scap-24*			0.41	n.d.	0.41	n.d.
CNS-35			0.36/0.85†	n.d./n.d.‡		
Sepi-37			0.22/0.5†	n.d./n.d.‡		
Scap-38*			0.26	n.d.	0.4	n.d.
Eco-42*			0.71/2.69†	n.d./n.d.‡		
Pmir-62			0.28/0.68†	n.d./w.b.‡		
Eco-64			0.11/0.54†	n.d./n.d.‡		

*Deceased.

#Subject 3, a separate bacteraemic episode.

†Two independent EDTA plasma collections within −30h<0<30h period relative to positive BC.

‡Two independent bacterial PCRs with five set of primers for each EDTA plasma collection.

Sepi - *S. epidermidis*; Scap - *S. capitis*; CNS - coagulase negative staphylococcus; Efu - *E*; *faecium*; Kpn - *K. pneumoniae*; Entc *- Enterococcus* spp; Psa - *P. aeruginosa*; Spn - *S*. *pneumoniae*; MRSA - methicillin resistant staphylococcus; MSSA - methicillin susceptible staphylococcus; Eco - *E. coli*; Pmir *- P. mirabilis*

### Stratification of bacteraemic subjects

**Fig. 4.**
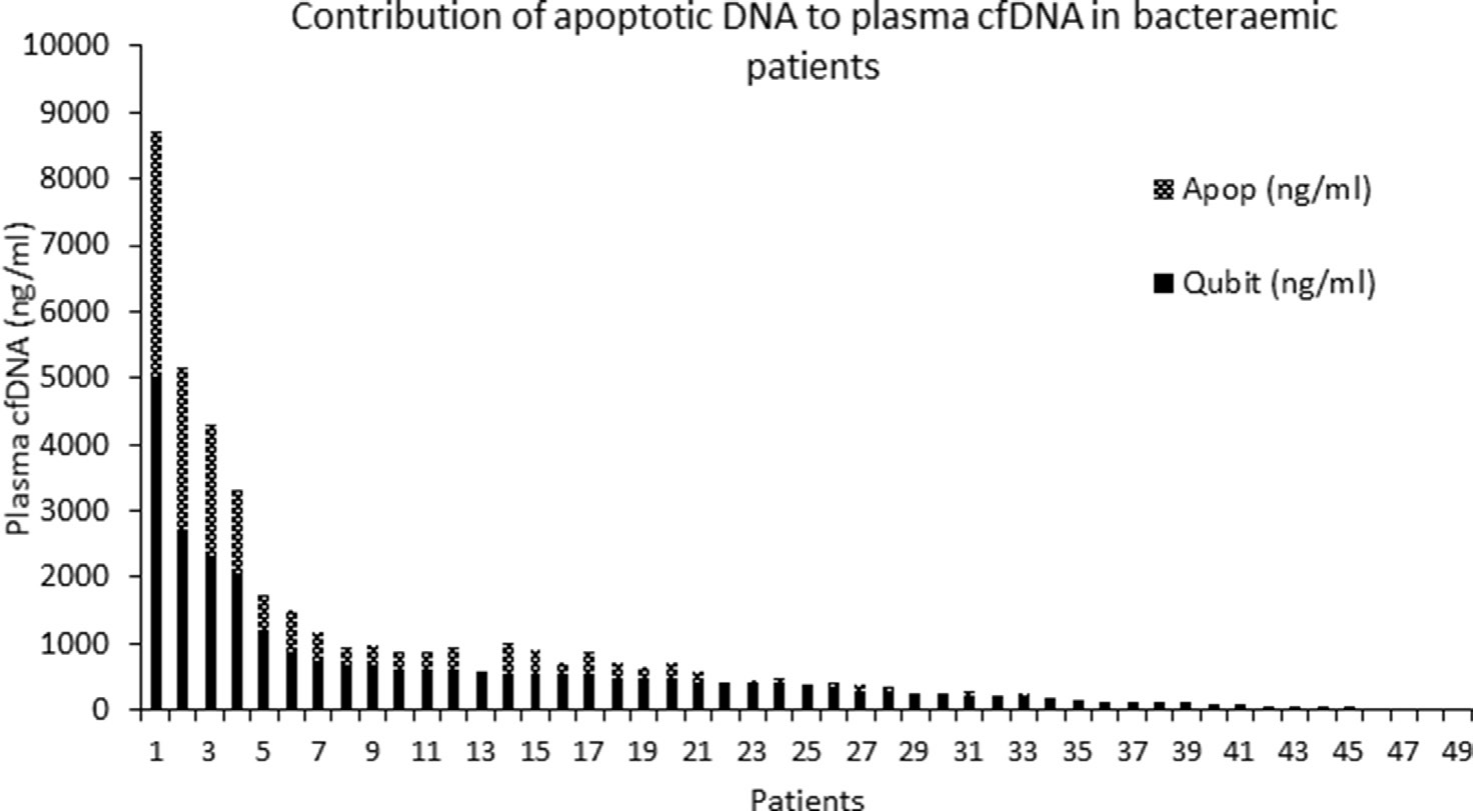
Apoptotic cfDNA distribution among bacteraemic subjects. Comparative analysis of total double stranded and apoptotic cfDNA in 49 bacteraemic subjects revealed an uneven distribution of apoptotic DNA among the subjects. In samples with high concentrations of total double stranded cfDNA the apoptotic DNA was also observed, while it was absent in the plasma of subjects with low amounts of total dsDNA [18]. There were 27 bacteraemic subjects out of 49 with detectable apoptotic DNA.

**Fig. 5.**
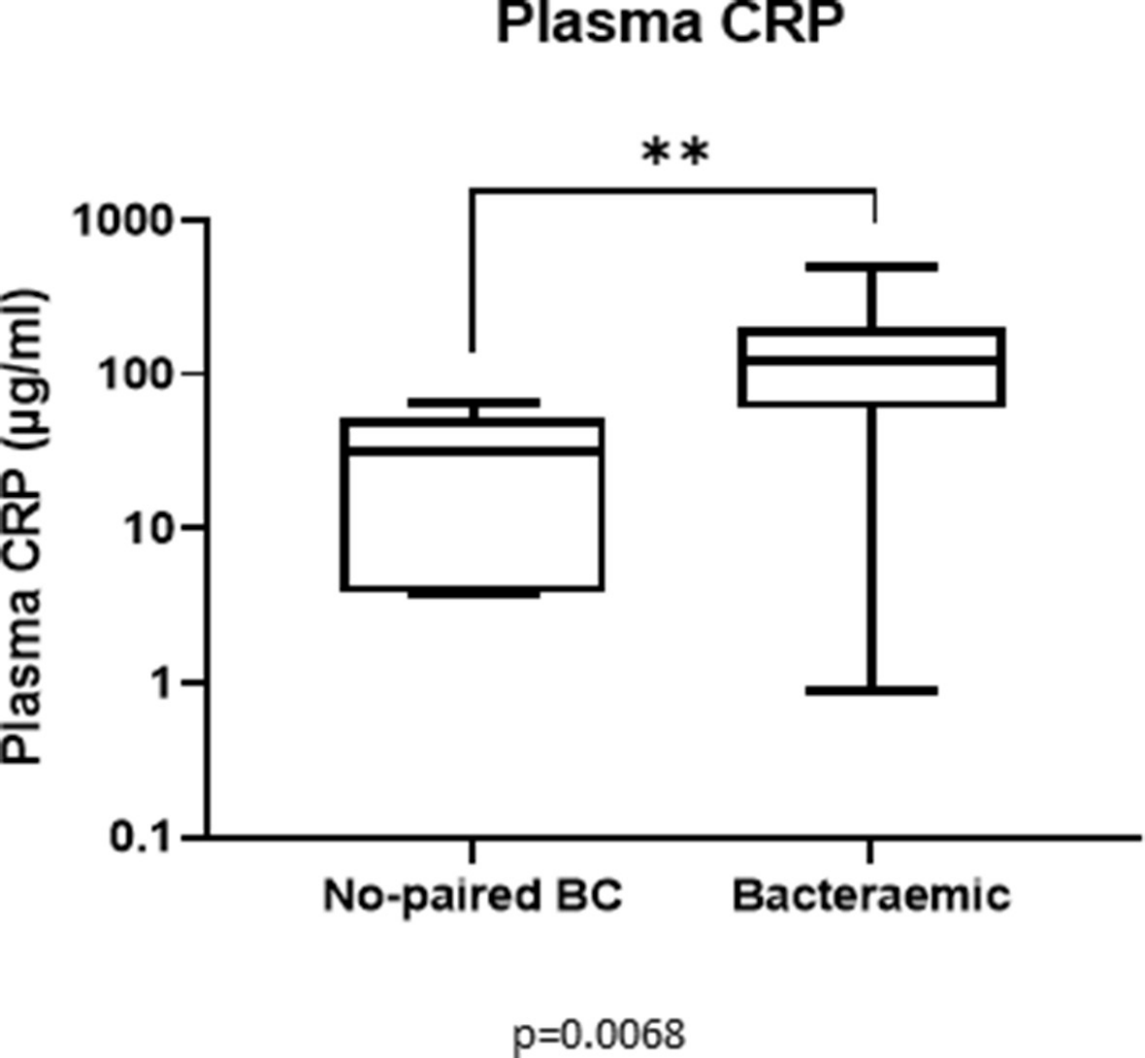
C-reactive protein (CRP) in hospitalised subjects. Both groups of hospitalised subjects, bacteraemic and no paired BC, had a CRP increased above the threshold value for healthy people (3–10 mg l^−1^). Bacteraemic subjects showed a further increase in comparison to no paired BC subjects (Mann Whitney U test, *P*=0.0068). The measurements were performed in the samples closest in time to BC collection.

Total dsDNA, nuclear and mitochondrial DNA were detected, although at significantly different levels, in both bacteraemic and no paired BC subjects and healthy controls while apoptotic DNA was present only in a subset of bacteraemic subjects and in none of healthy controls and no paired BC subjects. In more than 50 % of bacteraemic subjects, apoptotic DNA was the major cfDNA component in their plasmas (47–93 %), as shown in Fig. 4. A group of bacteraemic subjects had significantly higher levels of plasma CRP than no paired BC subjects (Fig. 5; Mann- Whitney U test, *P*=0.0068 and Fig. S3, *P*=0.014). When plasma CRP levels were further compared between two bacteraemic groups with and without apoptotic DNA, the bacteraemic subjects with apoptotic DNA had significantly higher plasma CRP than those without apoptotic DNA (170 µg ml^−1^ vs 84.5 µg ml^−1^; *P*=0.015 by Mann-Whitney two-tailed U test) consistent with a more severe clinical condition.

Comorbidities and survival between bacteraemic subjects with apoptotic cfDNA (BCA+) or without apoptotic cfDNA (BCA-) in plasma were then compared (Table 5). The comorbidities, underlying medical conditions, a subjects’ stay in the emergency department (ED) or intensive care unit (ICU), evidence of peripheral non-vital organ infections and suspected blood culture contamination are given in Table 5. Ten out of 27 bacteraemic subjects with apoptotic cfDNA (BCA+) died in hospital or were acutely ill in the ED or ICU, compared to none of the bacteraemic subjects without apoptotic cfDNA (BCA-). A further nine BCA +subjects had sepsis/SIRS/stroke/myocardial infarction or renal failure, none of which was observed in the BCA- subjects. In contrast, similar numbers of bacteraemic subjects in both groups (eight BCA+; six BCA-) had cancer, febrile conditions or surgical procedures, while 11 out of 22 BCA- and no BCA +subjects had peripheral organ or respiratory tract infections. There were five BCA- subjects with one of unconfirmed blood infection, missing medical records or outpatient treatment. Data in Table 5 show the association between the presence of apoptotic DNA in the plasma of bacteraemic subjects with complicated bloodstream infection, increased plasma CRP levels and WBC counts as well as with underlying comorbidities, in contrast to bacteraemic subjects with less severe outcomes of blood infection.

**Table 5.** Age, gender and comorbidities in bacteraemic subjects (BC) with (A+) or without (A-) apoptotic cfDNA in plasma

Subjects	BCA-	BCA+
No	22	27
Age (yr)*	59.2	68
Gender (F/M)	13/9	10/17
WBC (×10^3^ µl^−1^)	9.8	13
CRP (mg l^−1^)	102.7	196.7
Deceased	0	5
ED/ICU	0	5
Sepsis/SIRS	0	4
Stroke/Heart/Kidney	0	5
Cancer/Tumour	3	4
Febrile/Surgery	3	4
Chest/Respiratory	5	0
Periph Infection	6	0
Cont/Other†	5	0
Survivors (%)	100	81.5

*Age difference between BCA- and BCA+ *P*=0.06.

†Cont/Other – two subjects with suspected skin contamination, one outpatient subject and two with no available hospital histories.

## Discussion

Bloodstream infection is a potentially life-threatening medical condition in which bacteria play a decisive role. While laboratory-confirmed bloodstream infection per se is not sufficient to predict the development of a systemic clinical syndrome or ‘sepsis’, the poor correlation of laboratory parameters in early bacteraemic infection with disease progression and eventual outcome does not allow a cohesive laboratory definition of sepsis. Anticipating the onset of the clinical syndrome known as sepsis is a difficult task, but one that should be addressed to improve the quality and impact of treatment, and thus reduce sepsis mortality.

This study focused on subjects admitted to hospital with laboratory-confirmed bloodstream infections. Our aim was to develop blood plasma cfDNA extraction and analysis methods that would be easy to apply in a clinical laboratory for analysis of different (nuclear, mitochondrial, nucleosomal and microbial) cfDNA sub-types. This was the first time that all four DNA types were examined simultaneously in bacteraemic subjects. Previous studies either examined the ability of nuclear DNA, mostly *β globin* gene, to predict the outcome of infection [28–33] or, more recently developed metagenomic approaches to identify minute amounts of bacterial cfDNA in plasma of subjects with suspected bacteraemia using next generation sequencing (NGS) [34–36]. These two approaches independently studied the diagnostic utility of either human nuclear or bacterial chromosomal cfDNA in sepsis.

The former studies on nuclear plasma cfDNA in sepsis were performed in ICU and non-ICU settings [29, 30, 32, 33], in which increased plasma cfDNA, determined by either spectrophotometry or qPCR of the *β globin* gene, served as an independent predictor for ICU mortality [29, 30, 32, 33], but not for hospital mortality [30]. Increased plasma cfDNA levels were also reported in fatal cases of bacteraemia caused by four commonly isolated bacterial species in a study on 132 bacteraemic subjects at Tampere University Hospital in Finland [31]. In the same study, increased plasma CRP levels and apoptotic cfDNA in plasma of non-survivors was also reported [31], an observation later confirmed in the ICU sepsis subjects by others [32]. Our findings on plasma apoptotic DNA in bacteraemic subjects with increased plasma CRP levels and mortality are consistent with the earlier reports. The novelty was that we determined the concentration of apoptotic DNA demonstrating it was the major DNA component in the plasma of subjects with established bacteraemia (47 –93 % of total plasma cfDNA). In addition, the average apoptotic fragment size of 168 bp determined in this study indicated its local origin, mainly from apoptotic blood cells.

Thus far, there has not been supporting evidence for a specific source of cfDNA in bacteraemia or sepsis. As noted elsewhere [37], cellular DNA is released from the cells during necrotic or apoptotic cell death as naked DNA, vesicle-bound DNA or in macromolecular complexes with proteins or lipids. The main source of cfDNA in the peripheral circulation is in blood cells although traumatic tissue injury, cancer and organ transplantation can contribute to raised plasma cfDNA and thus affect its diagnostic application [38–40]. Programmed cell death or apoptosis yields fragmented nuclear DNA enriched in short fragments at or below 200 bp corresponding to a nucleosome; a structural unit of human chromosomes [18, 39, 41]. The circulating apoptotic cfDNA fragments above 150 bp and below 200 bp, that consisted of 146 bp nucleosome core and linkers, have previously been shown to originate from the hematopoietic cells, while DNA fragments shorter than 150 bp consisting of core DNA without linkers have been reported to derive from peripheral organs of non-hematopoietic origin due to a longer exposure to tissue nucleases [42]. The apoptotic cfDNA we observed in our study ranging from 160 bp to 192 bp (median 168 bp) consisted of the nucleosome core DNA of 146 bp plus 14 bp to 46 bp undigested linkers, suggesting a local blood cell origin.

We have also measured the mitochondrial DNA (mtDNA) that is a known constituent of neutrophil extracellular traps, together with nuclear DNA and proteins [37]. Plasma cell-free mtDNA has been implicated in some pathological conditions associated with increased inflammation such as acute respiratory distress syndrome, and has been linked to mortality in the ICU [43]. A recent review identified the potential use of mtDNA as a biomarker of disease severity and subsequent mortality in sepsis [44]. Our observation that mtDNA was non-selectively increased in all hospitalized subjects without preferential increase in bacteraemic subjects with more critical conditions strengthens the argument for further exploration of mtDNA in sepsis.

The presence of bacterial DNA in the whole blood and plasma has been previously documented in healthy individuals using PCR and metagenomics [45, 46]. While a proportion of bacteria is sequestered within blood cells, various amounts could be found free in circulation. In addition, bacterial cfDNA has been detected in circulation within membranous extracellular vesicles even in the absence of live bacteria [47]. The utility of bacterial DNA detection by PCR for predicting mortality in sepsis has been previously explored in the whole blood [48] as well as used in plasma of sepsis subjects with limited efficacy [49]. In our study, bacterial PCR with five sets of 16S primer pairs applied to 28 plasma specimens derived from 15 bacteraemic and one no-paired BC subjects had a very low utility revealing only a single positive signal in one specimen with one set of 16S primer pair. PCR may not provide the most sensitive diagnostic method for detecting bacterial cfDNA in plasma, as was reported for NGS and nanopore DNA sequencing [35–37]. The approach we developed could detect and measure human apoptotic, nuclear and mitochondrial DNA in 2–3 h after the blood draw. This could usefully complement the current NGS-based metagenomic approaches to bacterial DNA detection in plasma with significantly improved sensitivity and turnaround time [35, 36]. Recent reviews have indicated a growing interest in cfDNA application in cancer and non-cancer studies including sepsis [50], as well as its utility in predicting complicated COVID-19 [51].

In conclusion, this study provides support for plasma apoptotic DNA measurement as an indicator of disease progression in bacteraemic infection in hospitalized subjects. As the assays we used generate definitive results much earlier than traditional culture-based methods, there is a strong argument for their inclusion in the early assessment of patients with suspected bacteraemic infection. The best place to apply the tests we describe here is in emergency departments where rapid assays of plasma cfDNA might be used in the earliest stages of sepsis as a means of early detection of established bacteraemic infection and disease progression. Plasma cfDNA measurement may also provide a laboratory means to monitor sepsis progression and response to therapy and could serve as an objective alternative to the SOFA scoring system.

## Supplementary Data

Supplementary material 1
