# Supplementary material for: Plasma cfDNA predictors of established bacteraemic infection
Source: Access Microbiol. 2022 Jun 14;4(6):acmi000373. doi: 10.1099/acmi.0.000373 (PMC9394668; doi:10.1099/acmi.0.000373)
Supplement: Supplementary material 1 [file acmi-4-373-s001.pdf]

SUPPLEMENTARY MATERIAL

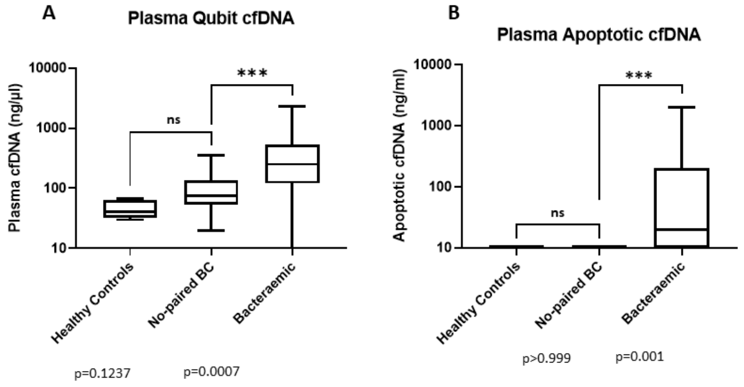

S1 Fig. Analysis of total plasma dsDNA and apoptotic DNA between different subject groups in EDTA blood samples paired to positive BC. A) The total amount of dsDNA extracted from bacteraemic subjects' plasma, no paired BC subjects and healthy controls was determined by Qubit. **B)** Following a microfluidic DNA gel electrophoresis of total plasma dsDNA, a discrete DNA band of ~165-180 base pairs was identified and measured in different groups of subjects and healthy controls by Bioanalyzer. The statistical analyses in A and B were performed with GraphPad Prism 9. Variance was determined by the Mann Whitney U test. EDTA blood samples the most proximal to BC were used in the analysis.

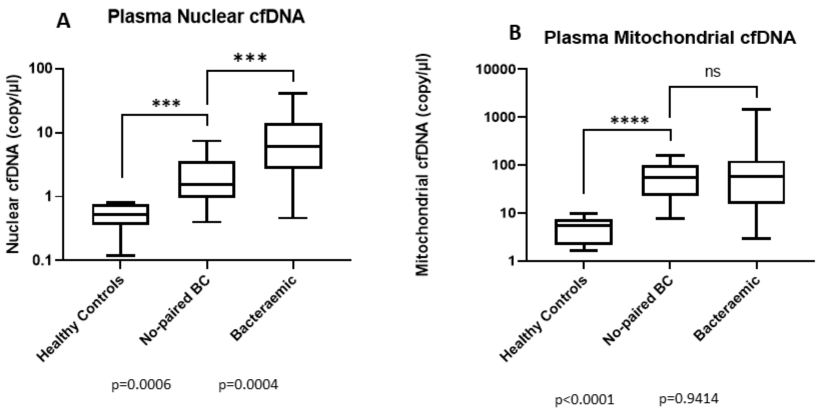

S2 Fig. Copy number of nuclear *β globin* and mitochondrial *MTATP8* genes by ddPCR in EDTA blood samples paired to positive BC. A) The copy number of the nuclear *β globin* gene in plasma cfDNA of healthy controls and two groups of hospitalised subjects, no paired BC and bacteraemic, shown here using box and whisker plots (GraphPad Prism 9). **B)** The copy number of the mitochondrial *MTATP8* gene was simultaneously determined for the same healthy controls and no paired BC/bacteraemic subjects and plotted using box and whisker plots (GraphPad Prism 9). Variance was determined by the Mann Whitney U test. EDTA blood samples most proximal to BC with the highest plasma CRP levels were used in the analysis. EDTA blood samples the most proximal to BC were used in the analysis.

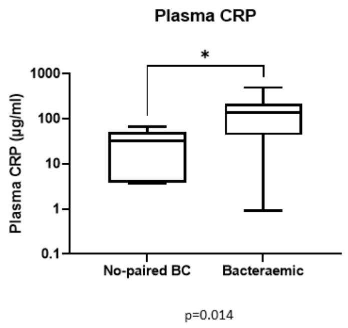

S3 Fig. C-reactive protein (CRP) in plasma paired to positive BC. Both groups of hospitalised subjects, bacteraemic and no paired BC, had a CRP increased above the threshold value for healthy people (3-10 mg/L). Bacteraemic subjects showed a further increase in comparison to no paired BC subjects (Mann Whitney U test, p=0.014). The measurements were performed in the samples paired to BC.
